# Gait training early after stroke with a new exoskeleton – the hybrid assistive limb: a study of safety and feasibility

**DOI:** 10.1186/1743-0003-11-92

**Published:** 2014-06-02

**Authors:** Anneli Nilsson, Katarina Skough Vreede, Vera Häglund, Hiroaki Kawamoto, Yoshiyuki Sankai, Jörgen Borg

**Affiliations:** 1 Department of Rehabilitation Medicine, Danderyd University Hospital, Building 39, floor 3, SE- 182 88 Stockholm, Sweden; 2 Department of Clinical Sciences Karolinska Institute, Stockholm, Sweden; 3 Faculty of Systems and Information Engineering, University of Tsukuba, Tsukuba, Japan

**Keywords:** Gait, Stroke, Rehabilitation, Robotics

## Abstract

**Background:**

Intensive task specific training early after stroke may enhance beneficial neuroplasticity and functional recovery. Impaired gait after hemiparetic stroke remains a challenge that may be approached early after stroke by use of novel technology. The aim of the study was to investigate the safety and feasibility of the exoskeleton Hybrid Assistive Limb (HAL) for intensive gait training as part of a regular inpatient rehabilitation program for hemiparetic patients with severely impaired gait early after stroke.

**Methods:**

Eligible were patients until 7 weeks after hemiparetic stroke. Training with HAL was performed 5 days per week by the autonomous and/or the voluntary control mode offered by the system. The study protocol covered safety and feasibility issues and aspects on motor function, gait performance according to the 10 Meter Walking Test (10MWT) and Functional Ambulation Categories (FAC), and activity performance.

**Results:**

Eight patients completed the study. Median time from stroke to inclusion was 35 days (range 6 to 46). Training started by use of the autonomous HAL mode in all and later switched to the voluntary mode in all but one and required one or two physiotherapists. Number of training sessions ranged from 6 to 31 (median 17) and walking time per session was around 25 minutes. The training was well tolerated and no serious adverse events occurred. All patients improved their walking ability during the training period, as reflected by the 10MWT (from 111.5 to 40 seconds in median) and the FAC (from 0 to 1.5 score in median).

**Conclusions:**

The HAL system enables intensive training of gait in hemiparetic patients with severely impaired gait function early after stroke. The system is safe when used as part of an inpatient rehabilitation program for these patients by experienced physiotherapists.

## Background

Stroke is a global health problem and one major cause of acquired disability in adults [1]. Hemiparesis is the most common acute manifestation of stroke and often impacts on gait function [2,3]. Although performance is improved in most stroke survivors during the first months post stroke, one third or more will need assistance in walking and remains limited in community ambulation [3,4]. Thus, independent gait remains a challenge in the rehabilitation after stroke [5].

Normal gait requires postural control, weight shifting and rhythmic and correct timing of muscle activity during repeated gait cycles and depends on the integrity of a complex interaction in sensory-motor neural networks at both spinal and supraspinal levels [6]. Depending on location and extent of the lesion and of restorative and compensatory mechanisms [7], gait characteristics may vary between and within patients over time after stroke.

Accumulating evidence indicates that early onset, intensive, repetitive task specific training may accelerate functional restitution after stroke and improve final motor outcomes [8], including gait function [9,10] although most motor rehabilitation trials have been performed in the chronic stage post stroke [11]. Even though early onset and intensive training of motor functions after stroke may enhance recovery by driving beneficial neuroplasticity a better understanding and prediction of the individual capacity and response to specific training paradigms remains a challenge [12,13].

Devices used to support gait training after stroke include treadmill training with or without body weight support (BWS). A recent Cochrane review found no overall statistically significant effect on gait function after treadmill training with BWS as compared with training without BWS [14] but this may differ between subgroups of patients with various severities and gait velocity during training may also have an impact [15]. These devices may be combined with electromechanical “gait machines”, which can allow more reproducible gait movements when compared to when a therapist move the patients legs. Gait machines are often categorized as machines using an end-effector principle and machines that function as exoskeletons [16]. Machines based on the end-effector principle use foot plates that move the feet in a controlled gait pattern and allow the operator to adjust many aspects of locomotion, such as speed, stride length and step height. In contrast, exoskeletons such as Lokomat [17] are attached to the patient and function as an external skeleton. Exoskeletons for lower extremities have joints matching the patient’s lower limb joints and motors that drive movements over these joints to assist leg movements. A recent Cochrane review concluded that electromechanical-assisted gait training in combination with physiotherapy after stroke increased the odds of participants becoming independent in walking and most so when this is applied in the first three months after stroke in patients, who are not able to walk [18]. However, further studies are needed with regard to the role of current types of electromechanical device [18,19] as well as to new concepts and devices and their evaluation in clinical trials [20,21]. One conceptual issue relates to the importance of incorporating active participation in the training. This has been approached in several studies by comparing training by use of gait machines such as Lokomat or Gait Trainer only, with regular therapist training that allows more variation, or combinations of these [22-28]. One new approach was taken by Krishnan et al. [29], who used a Lokomat device designed to allow patient cooperation and a target tracking task for matching of the ankle position to a movement template provided on a screen. A multilevel outcome analyses indicates that such active robotic training may be a potentially effective training model.

Recently, an exoskeleton with a hybrid system that allows both an automatic and a voluntary mode of action to support training of gait, the Hybrid Assistive Limb system (HAL) has been developed [30-32] and introduced in clinical trials [33-35]. This exoskeleton provides support according to the patient’s condition by a control algorithm and supporting devices, where each joint (left and right hip and left and right knee) can be controlled separately. The key features of the HAL system has been reported in detail previously [30-32] and is briefly outlined here.

The HAL system comprises two subsystems for (cybernic) voluntary control (CVC) and (cybernic) autonomous control (CAC) respectively. Both modes of action depend on the user’s intention in different ways. The CAC mode utilizes voluntary weight shift to initiate gait cycles and then provides predefined movements while gait in the CVC mode continuously use input from voluntarily activated gait muscles to provide support by the exoskeleton. This is achieved by recordings of the bioelectrical signals generated during muscle activation, as described by Kawamoto et al. 2002 [30]. Surface electrodes are placed over lower extremity extensor and flexor muscles and the recorded signals are incorporated in the control algorithm. The technology enables even weak muscle activity to be used to initiate and adjust the assistive torque, which may then be modified by a therapist [30]. Output is magnified and adjusted to the level of assistance needed over each hip and knee joint. A main controller of the system is used to control the power units, monitor the batteries, communicate with the system operator and modulate the assisting torque of each power unit. HAL is equipped with a sensing system receiving input also from potentiometers that are mounted on each joint and used as angular sensors to measure the joint angles, from force-pressure sensors in the shoes and from a gyro sensor and an acceleration sensor, which are mounted on the HAL body trunk, to measure posture [32].

The CVC mode allows the operator to adjust the degree of physical support for each joint and gradually reduce support as training progress. The EMG input*,* the adjusted torque limit and torque tuner for each joint and the adjusted assistance level for the flexor and extensor muscle groups respectively all together determine the power output [30]. These settings can not be standardized but are individually adapted over time. Settings are modified by the therapist during the training session depending on the patient’s performance in order to achieve a gait pattern that is as close as possible to normal gait. If the subject is paralytic, as may be the case early after stroke, the CAC mode may be used. Gait is then initiated and sustained by the voluntary locomotor intention, based on output from force-pressure sensors in the shoes. In this mode, the exoskeleton will e.g. swing the left leg when enough weight is put on the right leg in stance phase.

Gait training with HAL may be performed with or without BWS. Recently, aspects on feasibility and safety of HAL have been reported for early mobilization of patients in a neurosurgical ward by use of a prior version of the HAL system [35] and for gait training in patients with chronic impairment after a variety of conditions including stroke [33,34].

The aim of the present study was to explore the safety and feasibility of the HAL system when used for early onset, intensive gait training as part of an inpatient rehabilitation program for patients with hemiparetic stroke. Specifically, we wanted to explore the applicability of the system in patients with severely impaired gait function.

## Methods

### Patients

The trial was conducted at the Department of Rehabilitation Medicine, at Danderyd University Hospital and integrated in the individualized, team based, regular inpatient program. Eligible were patients living in the Stockholm region and who were admitted to the department for post-acute, inpatient rehabilitation after stroke between June 2012 and August 2013. Inclusion criteria were: less than seven weeks since stroke; able to sit on a bench with/without supervision at least five minutes; unable to walk independently due to lower extremity paresis with/without somato-sensory impairment and with/without spasticity; sufficient postural control to allow upright position in standing with aids and/or manual support; ability to understand training instructions as well as written and oral study information and to express informed consent; body size compatible with the HAL suit. Exclusion criteria were: contracture restricting gait movements at any lower limb joint (hip, knee, ankle); cardiovascular or other somatic condition incompatible with intensive gait training; severe, contagious infections (e.g. with Methicillin Resistant Staphylococcus Aureus (MRSA) or Extended Spectrum Beta-Lactamase (ESBL) bacteria). Eight consecutive patients fulfilled these criteria and completed the study protocol.

### Training program

**Figure 1:**
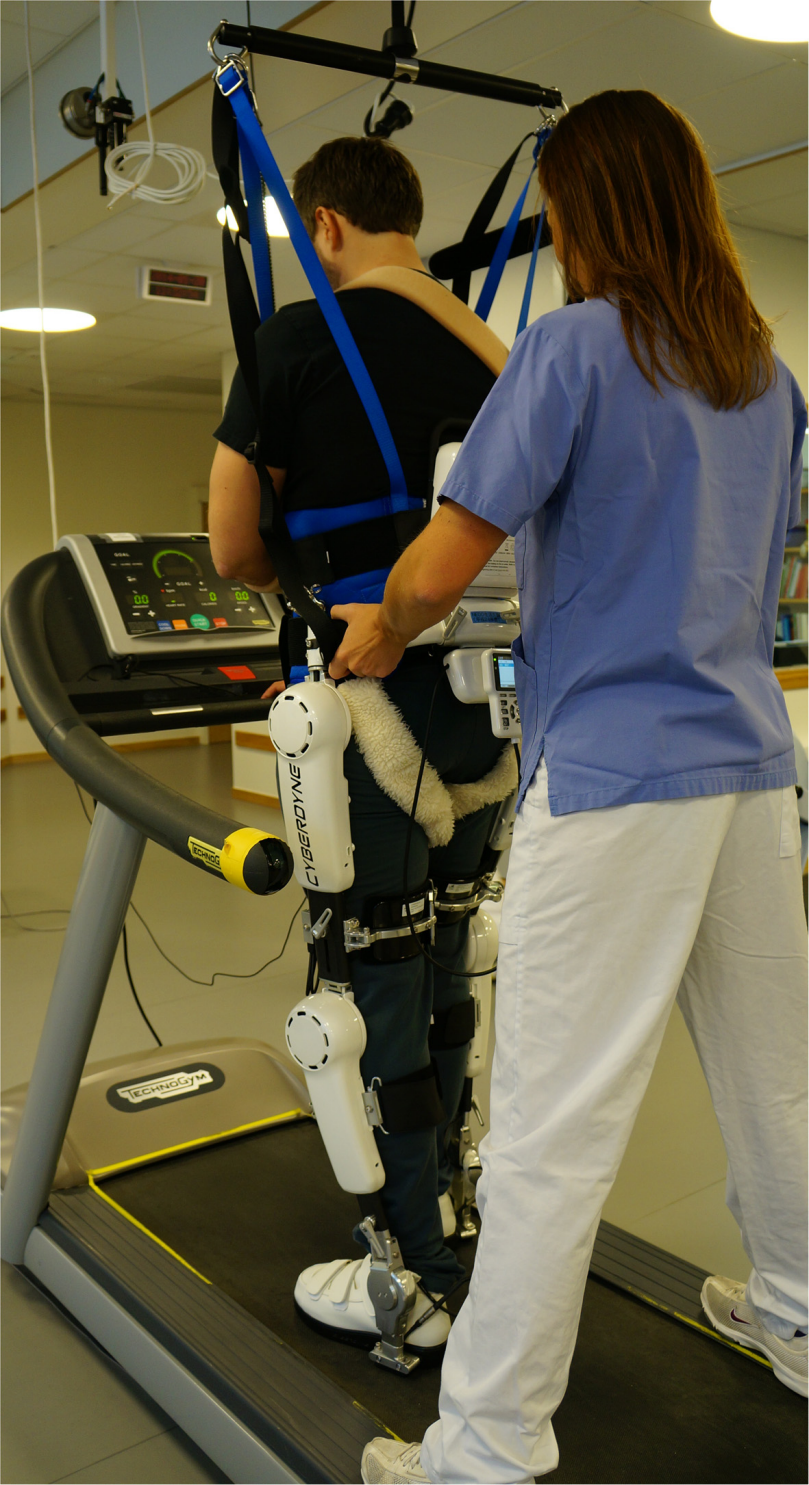
Illustration of Illustration of training training.

Training with HAL was performed during daily sessions on Monday to Friday. The patient was encouraged to walk as long time as he/she was able to including pauses, without exceeding 60 minutes (net walking time). In all patients, training with HAL was used in combination with BWS and treadmill for safety reasons and to allow adjustments of speed. The degree of BWS was individualized but never less than the weight of the HAL-suit, i.e. 14 kilos. Support from handrail of the treadmill was allowed. Training was performed by one or two physiotherapists (PT’s), who had learned and trained to use the HAL system (HAL-ML05). The double leg version was used and the suit was attached to the patients when standing or sitting. For an illustration of HAL training see Figure 1. The physiotherapist provided verbal instructions, encouragement and feedback to the patient. A mirror placed in front of the patient allowed visual feedback. Training started with the CAC and/or CVC mode for hip and knee joint on the affected side and aimed to use the CVC mode as soon as possible. HAL assistance was successively decreased as convenient according to the PT’s evaluation. Initial walking speed was 0.4 km/h, increased as tolerated and set at the highest speed possible to be compatible with the necessary HAL assist level. The degree of BWS was then successively reduced but adapted not to hamper optimal speed. All settings were individualized and adjusted to optimize normal gait pattern, which was evaluated through continuous observational gait analysis during training, according to the ten-point-checklist suggested by Kirtley [36]. The duration of the total treatment period was individualized. Training with HAL was stopped when HAL was no longer considered useful by the PT or when three months had elapsed since the stroke. Training with HAL was integrated in each participants individualized program according to current practice. This included goal oriented individualized and/or group training at the discretion of the rehabilitation team.

The study protocol addressed: (1) time to arrange the equipment and initiate a training session with HAL, (2) gait speed and quality at baseline and immediately after the training period, (3) utilization of BWS and of conventional aids such as orthoses during HAL training, (4) adverse events (such as falls, skin impact, pain etc.) related to use of HAL, technical risk factors and prevention of these, (5) patients attitudes towards training with HAL. In addition, training data (e.g. gait speed and distance) and HAL settings were registered at each training session.

### Outcome measures

Assessments at baseline and at endpoint comprised: the NIH Stroke Scale (NIHSS) [37]; the Fugl-Meyer Scale for the lower extremities (FM-LE) [38]; Bergs Balance scale (BBS) [39,40]; Timed Up and Go (TUG) [41]; 10 Meter Walking Test (10MWT) [42] self-selected and maximum speed; the Clinical Outcome Variable Scale, Swedish version (S-COVS) Section 5–8 [43]; Functional Ambulation Categories (FAC) [44]; Falls-efficacy Scale Swedish version (FES(S)) [45]; Barthel Index (BI) [46]; Functional Independence Measure (FIM) [47]; EQ-5D and EQ-5D VAS [48]. In addition, patient’s attitudes towards HAL training were captured by use of a visual analogue scale (VAS) ranging from 0 (negative) to 10 (positive). Further, relevant comments were documented during the training sessions. All assessments were conducted by the same physiotherapist who was not blinded to the intervention.

### Ethical approval

The study was approved by the Stockholm Ethical Review Board (Dnr: 2012/696-31/1).

### Clinical trial registration

The study was approved and registered as a clinical trial by the Swedish Medical Products Agency (Dnr: 461:2012/518333).

## Results

### Patients and injury characteristics

None of the patients who fulfilled study criteria declined participation and there were no drop-outs. All eight patients included were men. Median age was 56 years (range 39 to 64), median time from stroke to inclusion was 35 days (range 6 to 46). At baseline, Barthel Index ranged from 10 to 60 (median 30), FIM scores ranged from 26 to 96 (median 60). Demographic and injury characteristics are presented in Table 1.

**Table 1:** Patient characteristics and motor performance

**Case No.**	**Age**	**Type of stroke**	**Side of paresis (L/R)**	**Time from stroke to inclusion (days)**	**Number of training sessions**	**Net walking time/session (min) median (range)**	**Walking distance/session (m) median (range)**	**HAL mode of action**	**10MWT, self selected speed (s) B/E**	**10MWT, maximal speed (s) B/E**	**BBS (0–56) B/E**	**FAC (0–5) B/E**
1	64	H	L	46	20	21 (10–29)	231 (93–435)	CAC/CVC	−/−	−/−	4/5	0/1
2	39	H	R	46	16	22 (4–30)	403 (27–700)	CAC/CVC	147/32	147/15	5/37	1/2
3	55	I	R	11	16	45 (21–55)	813 (210–1188)	CAC/CVC	34/25	32/22	33/47	2/4
4	59	I	R	6	7	17 (7–18)	150 (70–300)	CAC	-/26	-/26	23/39	0/2
5	61	I	L	33	17	26 (7–44,5)	475 (82–1050)	CAC/CVC	480/244	480/244	7/10	0/1
6	48	I	R	39	31	32 (2–50)	533 (20–1125)	CAC/CVC	-/279	-/279	4/19	0/1
7	57	I	L	37	11	26 (9,5-32)	520 (127–675)	CAC/CVC	-/147	-/147	10/12	0/1
8	39	H	L	26	6	24 (20,5-46)	460 (376–920)	CAC/CVC	76/40	76/30	30/40	1/2

*Abbrevation*: *H* hemorrhage, *I* infarct, *L* left, *R* right, *CAC* cybernic autonomous control, *CVC* cybernic voluntary control, *B/E* Baseline/Endpoint, *10MWT* 10 Meter Walking Test, *BBS* Bergs Balance Scale, *FAC* Functional Ambulation Categories.

Data from assessments at baseline and immediately after finishing the training are presented in Tables 1 and 2. At baseline, FM-LE scores ranged from 41 to 63 (median 49) and gait was severely impaired as reflected by the TUG, 10MWT and FAC data (Table 2). Only one patient could perform the TUG test at baseline, 4 could perform the 10MWT and then needed on average 2 minutes, and the median FAC sore was 0 (Table 2). EQ5D and FES data are incomplete due to communication problems.

**Table 2:** Baseline and endpoint data, median (range)

**Measures**	**Baseline**	**n**	**Endpoint**	**n**
NIH stroke scale (0–42)	13 (7–18)	8	11 (5–14)	8
Fugl-Meyer, LE (0–86)	49 (41–63)	8	51 (39–68)	8
Bergs balance scale (0–56)	8.5 (4–33)	8	28 (5–47)	8
Timed up and go (s)	44 (44–44)	1	33.5 (24–42)	4
10 meter walking test, self selected speed (s)	111.5 (34–480)	4	40 (25–279)	8
10 meter walking test, maximal speed (s)	111.5 (32–480)	4	30 (15–279)	8
S-COVS, item 5–8 (4–28)	9 (4–16)	8	16.5 (8–20)	8
Functional ambulation categories (0–5)	0 (0–2)	8	1.5 (1–4)	8
Falls efficacy scale (0–130)	33.5 (3–67)	6	64 (24–96)	7
Barthel index (0–100)	30 (10–60)	8	55 (30–85)	8
Functional independence measure (18–126)	60 (26–96)	8	82 (45–106)	8
EQ-5D (Index)	0.015 (−0.043-0.639)	7	0.516 (−0.056-0.710)	7
EQ-5D VAS (0–100)	60 (40–70)	6	50 (10–98)	7

### Practical aspects on the use of HAL

HAL training was conducted by one or two PT’s, depending on the severity of the patient’s impairments. However, two therapists were present during donning and initial sessions for all patients and during the whole training period for 3 patients. A typical training session lasted around 90–105 minutes, including time for preparation of electrodes, putting on the harness and the suit and transfer to the treadmill. Time to arrange the equipment for training varied slightly depending on the patient's motor and cognitive skills. In general 15–20 minutes was needed from the patient arrived to the training session until gait training with HAL could start. Favorable factors were: ability to independently move between wheelchair and bench; ability to stand with the support of aids and moderate support of therapist when putting on for example electrodes, harness and HAL; ease to understand verbal instructions. Factors that increased the time for preparation were need for shaving the skin before attaching electrodes and need of great support in standing to maintain postural control. Time needed after finished HAL training (i.e. to remove the suit, harness and electrodes) was in general less than preparation time (around ten minutes). No patient needed a foot orthosis in addition to the support provided by the HAL suit and its connected shoe. All patients used the handrail unilateral (non paretic side) for support.

### Training program and clinical course

**Figure 2:**
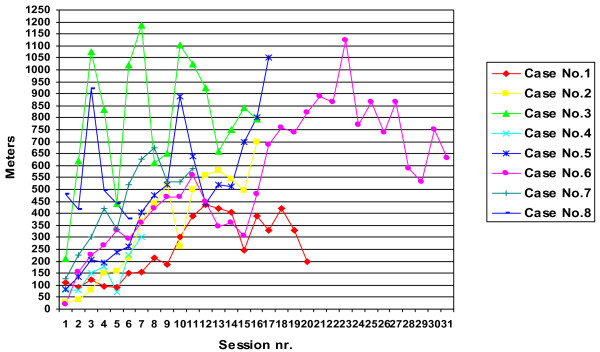
Walking distance by HAL training session.

Training data are presented in Table 1. Total, number of training sessions varied from 6 to 31 (median 16) depending on the clinical progress i.e. until HAL was no longer considered useful by the PT, or until three months after the stroke. The net walking time per session was around 25 minutes. The average individual walking distance per session ranged from 155 to 797 meters. The maximal walking distance observed during one session was 1188 meters in one patient (Case nr 3). Walking distances during HAL training for each study patient are presented in Figure 2. All patients started with the CAC mode and all except one later switched to use the CVC mode. On average, for these 7 patients, the initial 6 sessions out of 16 were with the CAC mode. The switch was performed as soon as the voluntarily induced EMG activity was sufficient to elicit a command signal. The amount of BWS provided was in median 27 (range 23–36) percent of the patients' body weight.

Measures of motor function, gait and of activity performance improved from baseline to the end of the training period in all patients (Table 2). Improvements in gait function in terms of FAC, increased by 1.5 units and time to walk 10 meters decreased by around 65% (n = 4). For further details see Table 2.

### Patient’s attitudes to use of the HAL system

All patients except one (with moderate communication problems) responded to the visual analogue scale. Overall, patients’ attitudes were positive. The average VAS rating of the “over all attitude to continue training with HAL”, was 7/10 (range 0.5-10).

### Adverse events

No serious adverse events occurred. Minor and temporary side effects comprised pain due to pressure from the cuff over the knee (n = 1; managed by changing the height of the cuff) and over the malleolus (n = 1; managed by changing the angle of the lower leg and foot joint); moderate discomfort of tight straps and the feeling of being trapped (n = 1), discomfort from shoulder straps (n = 2); sense of the suit being heavy over the lower back (n = 1); temporary skin irritation/redness from electrodes (n = 2; disappeared after finishing HAL training); moderate pain in the groin after a HAL training session (n = 1) (related to chafing from the harness); chafed feet due to wrong shoe size (n = 1) (manage by change of shoes); slight risk of stumbling due to impaired weight shifting occurred from time to time (support by two therapists was needed). One patient reported pain in the paretic arm during training. However, similar pain did also occur during other training sessions and thus was not specific for training with HAL. Issues related to technical problems were few and did not affect patient safety.

## Discussion

The main findings of this study are that the HAL system enables intensive, repetitive gait training in hemiparetic patients with severely impaired gait function early after stroke and that the system is feasible and safe when used as part of an inpatient rehabilitation program for these patients by experienced physiotherapists. Although this study does not allow any conclusions on the additional value of training with HAL with regard to recovery rate or final outcome as compared to other gait training programs, the findings may guide further studies in this respect.

This is the first application of the new Hybrid Assistive Limb system that allows both training of gait induced by weight shift (autonomous mode) and a gait pattern induced by the voluntary drive to walk, in contrast to other gait machines such as Lokomat [17], in patients with a hemiparesis early after stroke. It is to the best of our knowledge the first study of early onset of intensive gait training after stroke that utilizes this type and model of exoskeleton (HAL-ML05). Patients included in the study represented the more severe spectrum of patients with hemiparetic impairments after stroke. Accordingly, all eight patients initiated the training period by use of the autonomous HAL mode, which allowed the training to start earlier than otherwise possible. All except one patient later switched to training by use of the voluntary HAL mode. It should be pointed out that the voluntary activation pattern, as reflected by electromyography and translated to assisted movements, is by definition disturbed in the condition at study and thus requires corrections, which are part of the HAL system, and that the HAL settings are individually adapted by an experienced physiotherapist to achieve a gait pattern as close to normal as possible.

A typical training session lasted for about 100 minutes. The length of the training sessions, set to maximally 60 minutes net walking time, was predetermined based on clinical experience of what may be feasible and with regard to integration with the regular rehabilitation program. This time frame turned out to correspond well with what participants could manage, general fatigue being the most common limiting factor. Interestingly, the study patients walked in average approximately 444 m/session. The walking distance during HAL training sessions did not increase linearly over time but exhibited considerable intra-individual variation, which probably reflects increasing demands when the degree of assistance was reduced, fluctuations of the patients’ medical condition, mood and vitality as well as the learning curve.

Even though the study does not allow any conclusion, it was a consistent clinical impression that use of HAL enabled patients to achieve longer walking distances than would have been possible by regular gait training with or without BWS. Comparison in this respect with results from other studies using other training devices are hampered by differences with regard to inclusion criteria, time since stroke, training program and study design.

The test protocol captured a broad range of functional aspects. As expected, all patients exhibited improvements from baseline to endpoint assessments. These probably reflect time dependent, spontaneous recovery, which is mainly completed within the first ten weeks after stroke [49,50] as well as beneficial effects of the regular rehabilitation program, while an additional effect of the specific training with HAL cannot be disentangled and obviously was beyond the scope of this study. It may be noted that the observed improvement of functional gait as assessed by FAC, seemed more pronounced than may be explained only by time. FAC units increased by more than 1 unit (from a median value of 0 to 1.5, in mean by 1.25) in our small study sample of patients who started the HAL training around five weeks after stroke onset. In a longitudinal study by Kwakkel et al. [50] of 101 patients the corresponding figure for change of FAC was 1.1 units (in mean) over a 16 weeks period from stroke onset and this time dependent increase was most pronounced during the initial six weeks. Thus, even if our data do not allow any conclusion the observations lend some support to the interpretation that the improvements observed do not only reflect spontaneous recovery over time. Most previous gait training studies that report improvements of FAC differ considerably from our study with regard to study samples, baseline levels and study design [22,24-27]. One study by van Nunen et al. [28] offer data that may be used for a cautious comparison. That study compared the recovery of walking in non-ambulatory patients in the subacute phase after stroke. At baseline, in mean 62 days for 16 patients performing Lokomat + conventional training, and in mean 67 days for 14 patients performing conventional training, FAC was 1.50 and 1.00 respectively. At week 10, FAC had increased by 1.25 in the combined therapy group and by 1.29 in the other group. This is similar to the observations in our study. However, the FAC score baseline level was lower and training onset was earlier (median 35 days) in our study.

Training with HAL was performed by use of BWS and treadmill in all patients, which offered better control of safety and of gait speed. The combined use of BWS and HAL worked smoothly and no obstacles for such combined use were observed. The treadmill enabled accurate recording of walking speed and distance for each patient and training session. Although the width of the treadmill occasionally limited weight shifting, it never stopped a training session. The use of a harness seems essential to allow the HAL training to be safe and feasible for patients with severe paresis in the early stage after stroke.

Regular gait training for the study patients had likely been by use of treadmill and BWS and then most patients would probably have needed manual assistance by two or more physiotherapists to move the paretic leg and to assist weight shifting. However the potential benefits with regard to therapist time as compared to conventional gait training remains to be investigated and time spent to put on the suit and adjust settings must also be considered. This time was dependent on the patient’s general physical condition but seemed not related to spasticity, sensory impairments or motor performance of the upper extremity and diminished over time.

No serious adverse events occurred during training with HAL. Minor and temporary side effects comprised e.g. local pain, skin irritation and sense of heaviness of the lower back by the suit. It should be pointed out that even though participating patients had severe motor impairments all had regained sitting balance and were able to communicate with the therapist. In addition, the physiotherapists who conducted the training were experienced in rehabilitation after hemiparetic stroke and had been trained to use the HAL system. Minor technical issues occurred but did never impact on safety or the training schedule.

According to the questionnaire as well as face to face contacts, patients’ attitudes were generally positive to training with HAL. Regaining independent gait function is often considered a primary goal in stroke rehabilitation and it is reasonable to assume that the option to start training early by use of HAL may serve as a motivating factor.

### Study limitations

This prospective study is based on a small study sample at one study site and used no blinding or control group. The study included a selected subgroup of patients, who are not representative for the whole stroke population with regard to age, gender or neurological impairments. Notably, all study patients were men in spite of consecutive inclusion according to the study criteria. This probably is a random effect even though an uneven gender distribution (with around twice as many men) among patients, who are referred to the study clinic, may also have played a role. Thus, the findings are only relevant for the subgroup at study and cannot be generalized to the whole stroke population.

## Conclusions

This study of the Hybrid Assistive Limb system for intensive gait training early after stroke demonstrates that such training can be performed also by hemiparetic patients with severely impaired gait function and that the system is safe when used as part of an inpatient rehabilitation program for these patients by experienced physiotherapists. The observations should be useful for the design of further studies comparing training with the HAL system with other models for training of gait early after stroke.

## Competing interests

Anneli Nilsson, Katarina Skough Vreede, Vera Häglund and Jörgen Borg have no competing interests to declare. Hiroaki Kawamoto is a founder, shareholder, and an external director of CYBERDYNE Inc. which produces the HAL. Yoshiyuki Sankai is a founder, shareholder, and the CEO of CYBERDYNE Inc.

## Authors’ contributions

AN and KSV participated in the design of the study, carried out the HAL training and assessments, performed the statistical analysis and drafted the manuscript. VH included patients for participation in the study and collected their informed consent. HK and YS participated in the design of the study and provided the HAL suits and technical support. JB participated in the coordination and design of the study, in the statistical analysis and in drafting and finalizing the manuscript. All authors approved the manuscript.
